# Acknowledgement to the reviewers of GMS Journal for Medical Education

**DOI:** 10.3205/zma001443

**Published:** 2021-02-15

**Authors:** Martin R. Fischer, Götz Fabry

**Affiliations:** 1 GMS Journal for Medical Education, Chief Editor, Erlangen, Germany; 2 University Hospital, LMU Munich, Institute for Medical Education, Munich, Germany; 3 Albert-Ludwig-Universität Freiburg, Medizinische Fakultät, Abteilung für Medizinische Psychologie und Soziologie, Freiburg, Germany

## Acknowledgement

We gratefully acknowledge the valuable contribution of the following peer reviewers, who supported our Journal in 2020. We are looking forward to continuing our collaboration!

## Reviewers in alphabetical order

- Kristina Ackel-Eisnach, Landau
- Kambiz Afshar, Hannover
- Olaf Ahlers, Berlin
- Daniel Ammann, Chur, Switzerland
- Conny Herbert Antoni, Trier
- Jana Aulenkamp, Bochum
- Birgit Babitsch, Osnabrück
- Cadja Bachmann, Rostock
- Mahtab Bahramsoltani, Berlin
- Ivan Bank, Amsterdam, Netherland
- Anne Barzel, Ulm
- Daniel Bauer, Bern, Switzerland
- Johannes Bauer, Erfurt
- Andrea Baumann, Tübingen
- Jutta Begenau, Berlin
- Ronja Behrend, Berlin
- Marianne Behrends, Hannover
- Katrin Bekes, Vienna, Austria
- Pascal Berberat, Munich
- Alexandra Bergedick, Düsseldorf
- Birgit Berger, Hamburg
- Hendrik Berth, Dresden
- Stephan Birk, Berlin
- Eva Maria Bitzer, Freiburg i. Br.
- Vivian Blechschmidt, Heidelberg
- Sebastian Bode, Freiburg i. Br.
- Martin Boeker, Freiburg i. Br.
- Klaus Böhme, Bochum
- Katharina Bornkamm, Freiburg i. Br.
- Stefan Bösner, Marburg
- Barbara Braun, Heidelberg
- Jan Breckwoldt, Zürich, Switzerland
- Beate Brem, Bern, Switzerland
- Silja Brombacher-Steiert Hannover
- Heinz Hans Florian Buchner, Vienna, Austria
- Till Bugaj, Heidelberg
- Holger Buggenhagen, Mainz
- Andreas Burger, Bochum
- Jean-Francois Chenot, Greifswald
- Irmgard Claßen-Linke, Aachen
- Kevin Dadaczynski, Fulda
- A.J. de Beaufort, Maastricht, Netherland
- Saskia Degani, Zürich, Switzerland
- Iris Demmer, Göttingen
- Martin A. Denvir, Edinburgh, UK
- Kim Deutsch, Mainz
- Oliver Dietz, Mainz
- Andreas Dinkel, Munich
- Carsten Döing, Düsseldorf
- Karolin Dospil, Munich
- Simon Drees, Berlin
- Sabine Drossard, Munich
- Stephan Düsterwald, Hildesheim
- Friedrich Edelhäuser, Witten
- Jan P. Ehlers, Witten
- Simone Ehrhard, Bern, Switzerland
- Maren Ehrhardt, Hamburg
- Tobias Eichinger, Zürich, Switzerland
- Barbara Eichner, Ulm
- Christian Emsden, Basel, Switzerland
- Rebecca Erschens, Tübingen
- Mona Eulitz, Witten
- Götz Fabry, Freiburg i. Br.
- Nora Faubl, Pécs, Hungary
- Folkert Fehr, Sinsheim an der Elzenz
- Maximilian Fink, Munich
- Martin Fischer, Munich
- Volkhard Fischer, Hannover
- Carola Fischer-Tenhagen, Berlin
- Andreas Fleischer, Bonn
- Nina Fleischmann, Fulda
- Julia Freytag, Berlin
- Angelika Hiroko Fritz, Essen
- Martin R. Fröhlich, Aarau, Switzerland
- Annette Fröhmel, Düsseldorf
- Katharina Fürholzer, Ulm
- Martin Gartmeier, Munich
- Kirsten Gehlhar, Oldenburg
- Sabine Gehrke-Beck, Berlin
- Waltraud Georg, Berlin
- JarisGerup Herlev, Denmark
- Trevor John Gibbs, Dundee, UK
- Marianne Giesler, Freiburg i. Br.
- Heidrun Golla, Cologne
- Maryna Gornostayeva, Mainz
- Katja Götz, Lübeck
- Heide Götze, Leipzig
- Matthäus Grasl, Vienna, Austria
- Jan Griewatz, Tuebingen
- Katharina Günther, Leipzig
- Sissel Guttormsen, Bern, Switzerland
- Stefan Gysin, Luzern, Switzerland
- Martin Haag, Heilbronn
- Rainer Haak, Leipzig
- Petra Hahn, Freiburg i. Br.
- Eckhart G. Hahn, Erlangen
- Marietta Handgraaf, Bochum
- Sigrid Harendza, Hamburg
- Roman Hari, Bern, Switzerland
- Anja Härt, lAusburg
- Ute Hauck, Halle
- Sara Häusermann, Zürich, Switzerland
- Wolf Hautz, Bern, Switzerland
- Inga Hege, Augsburg
- Susanne Heim, Göttingen
- Felix Heindl, Ulm
- Gunther Hempel, Leipzig
- Linn Hempel, Düsseldorf
- Doreen Herinek, Berlin
- Wolfram J. Herrmann, Berlin
- Anne Herrmann-Werner, Tübingen
- Sonja Heuser, Munich
- Sybille Hildenbrand, Tübingen
- Monika Himmelbauer, Vienna, Austria
- Barbara Hinding, Mainz
- Birgit Hladschik-Kermer, Vienna, Austria
- Matthias Hofer, Bern, Switzerland
- Anke Hollinderbäumer, Mainz
- Henrike Hölzer, Berlin
- Ylva Holzhausen, Berlin
- Christopher Holzmann-Littig, Munich
- Angelika Homberg, Mannheim
- Anna Horrer, Munich
- Marion Huber, Winterthur, Switzerland
- Johanna Huber, Munich
- Florentine Hüttl, Mainz
- Sören Huwendiek, Bern, Switzerland
- Peter Iblher, Fehmarn
- Fabian Jacobs, Munich
- Paul Jansen, Kamen-Heeren
- Nana Jedlicska, Munich
- Jana Jünger, Mainz
- Florian Jungmann, Mainz
- Stephanie Jungmann, Mainz
- Sylvia Kaap-Fröhlich, Zürich, Switzerland
- Hanna Kaduszkiewicz, Kiel
- Canar Kamisli, Bochum
- Yassin Karay, Cologne
- Friederike Kendel, Berlin
- Michaela Key, Zürich, Switzerland
- Claudia Kiessling, Witten
- Christin Kleinsorgen, Hannover
- Andreas Klement, Halle
- Michael Knipper, Giessen
- Sabrina Koh Bee Leng, Singapore
- Sarah König, Würzburg
- Sabine Korek, Leipzig
- Jasmin Körner, Ulm
- Thomas Kötter, Lübeck
- Marc Krüger, Münster
- Daniela Kugelmann, Munich
- Susanne Kühl, Ulm
- Sebastian Kuhn, Bielefeld
- Sandy Kujumdshiev, Leipzig
- Evelyn Kunschitz, Vienna, Austria
- Miriam Kunz, Augsburg
- Jörg P. Kupfer, Giessen
- Rahel Kurpat, Münster
- Sandra Kurz, Mainz
- Nathalie Laissue, Basel, Switzerland
- Johannes Lang, Giessen
- Katharina Langer-Fischer, Ulm
- Wolf Langewitz, Basel, Switzerland
- Stefan Leis, Salzburg, Austria
- Andrea Lenes, Aachen
- Holger Lenz, Munich
- Teresa Loda, Tübingen
- Christin Löffler, Rostock
- Henriette Löffler-Stastka, Vienna, Austria
- Mark Lüdde, Bremen
- Clemens Ludwig, Halle
- Sabine Ludwig, Berlin
- Björn Lütcke, Erlangen
- Cornelia Mahler, Tübingen
- Karoline Malchus, Bielefeld
- Georg Marckmann, Munich
- Jörg Marienhagen, Augsburg
- Gabriella Marx, Hamburg
- Gregor Massoth, Bonn
- Johanna Mink, Heidelberg
- Sonja Mohr, Hamburg
- Andreas Möltner, Heidelberg
- Sören Moritz, Cologne
- Katja Anne Moschberger, Berlin
- Christiane Müller, Göttingen
- Brigitte Müller-Hilke, Rostock
- Christiane Muth, Frankfurt/Main
- Kai Niebert, Zürich, Switzerland
- Christoph Nikendei, Heidelberg
- Dino Carl Novak, Berlin
- Thomas Nowak, Mainz
- Heidi Oberhauser, Innsbruck, Austria
- Christian Offergeld, Freiburg i. Br.
- Thomas Ott, Mainz
- Stefanie Otten, Düsseldorf
- Alexander Palant, Berlin
- Robert Patejdl, Rostock
- Volker Paulmann, Hannover
- Michael Pentzek, Düsseldorf
- Tim Peters, Bochum
- Serge Petralito, Zürich, Switzerland
- Severin Pinilla, Bern, Switzerland
- Bettina Pollok, Düsseldorf
- Gominda G.Ponnamperuma, Nugegoda, Sri Lanka
- Ingrid Preusche, Vienna, Austria
- Reinhard Putz, Munich
- Alexander Rahman, Hannover
- Tobias Raupach, Göttingen
- Florian Recker, Bonn
- Kathrin Reichel, Berlin
- Matthias Reinhard, Munich
- Reimer Riessen, Tübingen
- Katrin Rockenbauch, Leipzig
- Bernd Romeike, Rostock
- Caroline Rometsch-Ogioun El Sount, Tübingen
- Marco Roos, Erlangen
- Eva-Maria Rosenmayr-Khemiri, Wiener Neustadt, Austria
- Thomas Rotthoff, Augsburg
- Stefan Rüttermann, Frankfurt
- Carmen Salatsch, Bonn
- Christina Sauer, Heidelberg
- Thomas C. Sauter, Bern, Switzerland
- Thorsten Schäfer, Bochum
- Robert Schafnitzel, Ulm
- Elisabeth Schaper, Hannover
- Christian Scheffer, Witten
- Jörg Schelling, Martinsried
- Christian Schirlo Luzern, Switzerland
- Anita Schmidt, Erlangen
- Martina Schmitz, Münster
- Kai Schnabel, Bern, Switzerland
- Antonius Schneider, Munich
- Achim Schneider, Ulm
- Andrea Schönbauer, Marburg
- Teresa Schreckenbach, Frankfurt
- Torsten Schröder, Berlin
- Rudolf Schubert, Augsburg
- Ina Schüler, Jena
- Andreas Schulte, Witten
- Katrin Schüttpelz-Brauns, Mannheim
- Tanja Schwarzmeier, Munich
- Eva-Maria Schwienhorst, Würzburg
- Kerry Shephard Dunedin, New Sealand
- Thomas Shiozawa-Bayer, Tübingen
- Anne Simmenroth, Würzburg
- Andreas Söhnel, Greifswald
- Ulrike Sonntag, Berlin
- Beat Sottas, Bourguillon, Switzerland
- Verena Steiner-Hofbauer, Vienna, Austria
- Jost Steinhäuser, Lübeck
- Sylvère Störmann. Munich
- Christoph Stosch, Cologne
- Frank Stümpel, Münster
- Alexander T. Teichmann, Aschaffenburg
- Nils Thiessen, Bielefeld
- Christian Thrien, Cologne
- Leonhard Thun-Hohenstein, Salzburg, Austria
- Andrea Tipold, Hannover
- Daniel Tolks, Munich
- Harald Tschernitschek, Hannover
- Judith Ullmann-Moskovits, Frankfurt am Main
- Gert Ulrich, Zürich, Switzerland
- Ivo van den Berk, Emden
- Barbara Vogel, Berlin
- Thomas von Lengerke, Hannover
- Jörn von Wietersheim, Ulm
- Michaela Wagner-Menghin, Vienna, Austria
- Ursula Walkenhorst. Osnabrück
- Regina Waury-Eichler. Berlin
- Rainer Weber, Cologne
- Johannes Weimer, Münster
- Birgit Wershofen, Munich
- Bärbel Wesselborg, Düsseldorf
- Mario Wijnen-Meijer, Munich
- Alexandra Wirth, Zürich, Switzerland
- Boris Wittekindt, Frankfurt/Main
- Ulrich Woermann, Bern, Switzerland
- Jan Zottmann, Munich
- Michaela Zupanic, Witten

